# Clinical Value of Bronchoscopy in Acute Respiratory Failure

**DOI:** 10.3390/diagnostics11101755

**Published:** 2021-09-24

**Authors:** Raffaele Scala, Luca Guidelli

**Affiliations:** Pulmonology and Respiratory Intensive Care Unit, S. Donato Hospital, Via Nenni, 52100 Arezzo, Italy; lucaguido@live.it

**Keywords:** bronchoscopy, acute respiratory failure, FBO

## Abstract

Bronchoscopy may be considered the “added value” in the diagnostic and therapeutic pathway of different clinical scenarios occurring in acute respiratory critically ill patients. Rigid bronchoscopy is mainly employed in emergent clinical situations due to central airways obstruction, haemoptysis, and inhaled foreign body. Flexible bronchoscopy (FBO) has larger fields of acute applications. In intensive care settings, FBO is useful to facilitate intubation in difficult airways, guide percutaneous dilatational tracheostomy, and mucous plugs causing lobar/lung atelectasis. FBO plays a central diagnostic role in acute respiratory failure caused by intra-thoracic tumors, interstitial lung diseases, and suspected severe pneumonia. “Bronchoscopic” sampling has to be considered when “non-invasive” techniques are not diagnostic in suspected ventilator-associated pneumonia and in non-ventilated immunosuppressed patients. The combined use of either noninvasive ventilation (NIV) or High-flow nasal cannula (HFNC) with bronchoscopy is useful in different scenarios; the largest body of proven successful evidence has been found for NIV-supported diagnostic FBO in non-ventilated high risk patients to prevent and avoid intubation. The expected diagnostic/therapeutic goals of acute bronchoscopy should be balanced against the potential severe risks (i.e., cardio-pulmonary complications, bleeding, and pneumothorax). Expertise of the team is fundamental to achieve the best rate of success with the lowest rate of complications of diagnostic and therapeutic bronchoscopic procedures in acute clinical circumstances.

## 1. Introduction 

Bronchoscopy was firstly introduced in 1897 for an emergent removal of an inhaled foreign body (FB). Since then, bronchoscopy use has been tremendously increased for several diagnostic and therapeutic purposes in patients admitted to respiratory high-dependency care units (RHDCU) and intensive care units (ICU) [1,2,3,4]. 

Bronchoscopy may be done with flexible and rigid instruments. Flexible bronchoscopy (FBO) is more widely employed due to its less invasiveness, deeper capability for exploration of bronchial tree, and quicker learning curve; its “ancillary techniques” allow sampling to be taken from lung and mediastinum, such as broncho-alveolar lavage (BAL), protected-specimen brush (PSB), trans-bronchial needle aspiration (TBNA), and trans-bronchial lung biopsy (TBLB) [1,2,4]. Rigid bronchoscopy (RB) has a more limited field of applications, but represents a mandatory safe and effective technique to perform interventional procedures, such as ablative treatments (laser, argon-plasma, electro-cautery, cryotherapy), airway’s stenting, as well as FBs removal [4]. 

The aims of this paper are to make an updated revision on the clinical indications for bronchoscopy in adults with acute respiratory failure (ARF), the patho-physiologic pitfalls of bronchoscopy in non-ventilated and ventilated patients, as well as on the role of noninvasive ventilation (NIV) and/or high-flow nasal cannula oxygen (HFNC) in “assisting” high-risk patients during bronchoscopic procedures. 

## 2. Clinical Indications of Acute Bronchoscopy

**Figure 1:**
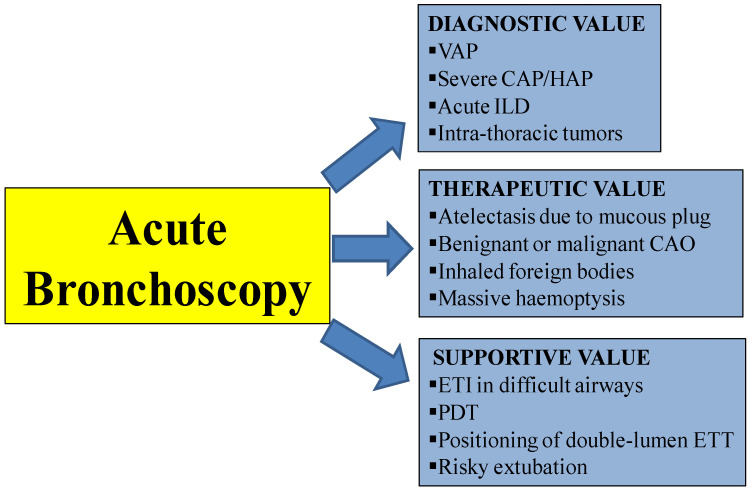
Clinical “added” value and indications of acute bronchoscopy. CAP = Community acquired pneumonia; CAO = central airway obstruction; ETI = Endotracheal intubation; HAP = Hospital acquired pneumonia; ILD = Interstitial lung disease; PDT = percutaneous dilatational tracheostomy; VAP = ventilator associated pneumonia.

The main indications for bronchoscopy in ARF patients are: (1) to support difficult intubation and percutaneous dilatational tracheostomy (PDT) [5,6,7]; (2) to help in the diagnosis of infective and non-infective lung infiltrates in non-ventilated and ventilated patients [1,8,9,10,11]; (3) to manage acute airway obstruction of different origins, such as mucous plugs, FBs, and benign/malignant trachea-bronchial disorders [3,12]; (4) to manage massive haemoptysis [13] (Figure 1).

### 2.1. Pneumonia 

FBO is not routinely indicated in non-ventilated acute patients with suspected or known severe community-acquired (CAP) and hospital-acquired pneumonia (HAP). This is due to the fact that peri-procedural risks could be higher than the expected diagnostic advantages correlated to the identification of the etiologic micro-organism(s). However, early BAL and or PSB may be of clinical usefulness in immune-suppressed patients with lung infiltrates and/or in pneumonia caused by multi-resistant microorganisms [1,8,9]. In other words, the likelihood of getting from acute bronchoscopy diagnostic findings that may impact on changes of treatment and possibly on the outcome of patients admitted to hospital for suspected severe CAP is higher for immuno-compromised hosts (i.e., infection due to opportunistic germs), lack of response to empirical antibiotic-therapy (i.e., unusual pathogens such as fungal and viral infections, multi-drug resistant bacteria), conditions mimicking infective pneumonia (i.e., severe organizing pneumonia, acute eosinophilic pneumonia, and drug-induced lung damage). 

In patients with suspected ventilator-associated pneumonia (VAP) undergoing invasive mechanical ventilation (IMV), respiratory tract sampling is recommended before the introduction/change of antibiotics, with the exception of some specific situations (i.e., severe sepsis, multi-organ dysfunction, shock), in whom antibiotic-therapy should not be delayed and have to be started empirically, followed by de-escalation strategy driven by microbiological results [14,15,16,17,18,19,20,21,22,23,24,25,26,27]. Bronchoscopic (e.g., BAL and/or PSB) and non-bronchoscopic samples (e.g., tracheo-bronchial aspiration or mini-BAL) are, respectively, the “invasive” “non-invasive” techniques applicable in patients with suspected VAP [14,15,16]. A cutoff in terms of bacterial growth count may discriminate between infection and colonization (i.e., PSB: 10^3^ cfu/mL; BAL: 10^4^ cfu/mL; endotracheal aspirates: 10^5^ cfu/mL) according to the “quantitative” analysis, while the presence or absence of pathogenic germs is enough for the “qualitative” analysis [2]. The sensitivities of bronchoscopic techniques vary largely between 51 and 100% in uncontrolled trials depending on a great heterogeneity in terms of gold standard for diagnosis, microbiological thresholds, employed techniques, and variable comparison to “non-invasive” techniques [1,14,15,16,17,18,19,20,21,22,23,24,25,26,27]. A recent systematic review [16] including five RCTs analyzed a total of 1367 immuno-competent patients with suspected VAP; no significant differences merged in terms of mortality rates, variation of antibiotic choices, and duration of IMV and of ICU stay between either “quantitative” versus “qualitative” analysis or “invasive” versus “non-invasive” sampling diagnostic methods. According to the accumulated published data, the choice of diagnostic FBO has to be individually made case per case and if “non-invasive” techniques fail to identify a responsible organism [2]. There is not enough evidence in the literature to give recommendations for the use of bronchoscopy in patients with severe CAP who subsequently require ventilation [1].

### 2.2. Acute Interstitial Lung Diseases

FBO with BAL play an important role for the challenging differential diagnosis that the clinician has to face in interstitial lung diseases (ILDs) presenting with ARF. The clinical value of bronchoscopic procedures is greater in the suspect of pulmonary infection occurring in either acute deterioration of a previously diagnosed ILD or in the rapid onset of “de novo” acute ILD [10]. This is especially true in high-risk conditions associated with ILDs such as neutropenia, immune-suppressive treatment, and onco-hematologic diseases [1,2,28]. In fact, BAL is likely to discriminate between infective exacerbations and “acceleration” of IPF or other ILDs [10,29,30,31]; thus this may be of help for the physician to solve out the ethical dilemma correlated to the choice of withholding ventilator support in subjects developing severe ARF or withdrawing mechanical ventilation in terminally ill ventilator dependent patients.

TBLB is more likely to provide an additional diagnostic value of over BAL in selected categories of ILD patients presenting with ARF, such as immune-suppressed conditions, granulomatous diseases (i.e., sarcoidosis) and lymphangitis carcinomatosis; instead, the role of TBLB in acute patients with idiopathic ILDs is uncertain in terms of balance between benefits against risks [1,10,32,33,34,35]. In non-intubated patients, the rate of complications (e.g., pneumothorax, hemorrhage) correlated to TBLB is relatively low, being less than <5% as compared to the perioperative risks of surgical lung biopsy (SLB). Conversely, implications of pneumothorax following TBLB in intubated and ventilated patients are by far more severe especially in terms of blood gases worsening [35,36]. Therefore, TBLB should be cautiously considered in very selected IMV patients. 

Transbronchial lung cryobiopsy (TBLC) has been recently introduced for the diagnosis of stable ILD providing larger and better preserved samples as compared to TBLB, with the risk of complications being between SLB and TBLB [37,38,39]. However, data confirming the potential benefits against the risks concerning the use of TBLC in ARF patients are lacking [2,37,38,39]. 

In severe acute hypoxemic ILD patients, BAL, TBLB, and TBLC under the noninvasive support given by HFNC or NIV are feasible and safe if performed in a protected environment where severe complications could be quickly treated [2].

### 2.3. Intra-Thoracic Tumors

Blood gas exchange’s derangement, central airway obstruction (CAO), life-threatening bleeding, superior vena cava syndrome, and extra-pulmonary metastases represent high risk conditions in critically ill patients undergoing FBO for the diagnosis and staging of intra-thoracic tumors [1,12,40].

Cardiopulmonary comorbidity diseases may increase the likelihood of peri-procedural hypoxemia, while the use of anti-coagulant and/or anti-platelets therapy is associated with greater occurrence of bleeding [1]. NIV or HFNC assisted bronchoscopy could be a safe strategy in severely hypoxemic neoplastic patients with/without hypercapnia requiring diagnostic and therapeutic interventional procedures [2].

The incidence of peri-procedural complications of FBO in intubated and invasively ventilated with suspected lung cancer is dependent on the clinical patient’s conditions, the gap between the size of bronchoscope and that of the endotracheal tube (ETT), and the complications of bronchoscopic procedures [1]. As reported above, TBLB may be associated with severe complications in IMV patients. In the opposite, lymph node sampling by means of TBNA is less risky [41]. Endobronchial ultrasound (EBUS)-TBNA is feasible in ventilated patients and provides a higher diagnostic yield in comparison to “blind” TBNA [42].

### 2.4. Haemoptysis 

Massive haemoptysis leading to failure of lung gas exchange and/or haemodynamic instability represents an emergency and is associated with a death incidence between 7 and 80% [13]. There is no agreement for the optimal diagnostic/therapeutic approach to manage life-threatening endobronchial bleeding. The diagnostic evaluation of patients with massive hemoptysis is focused on the localization of the bleeding site and on the detection of the underlying cause. Bronchoscopy is indicated for patients needing airway control and those in whom CT imaging cannot localize the bleeding site (e.g., bilateral lung disease). The treatment of severe endobronchial bleeding differs depending on the need of airway protection and on the hemodynamic status of the patient [43,44,45].

In patients with hemodynamic stability, good airway control, and adequate blood gases, the identification of the bleeding source can be performed quickly with CT imaging. The more widespread availability of bronchial artery embolization has led to a shift in the management of life-threatening hemoptysis towards an early radiologic interventional procedure. FBO is indicated when CT fails to localize the bleeding site and/or detect the reason of the haemoptysis. The capability of bronchoscopy in finding the site of bleeding and blocking endobronchially haemoptysis is variable and depends on the different clinical conditions [43,44,45]. In case of peripheral bleeding, diagnostic bronchoscopy is indicated to identify the site of the bleeding and to drive either radiological interventional procedures with bronchial arterial coils or surgical treatment. 

In the case of massive bleeding leading to haemodynamic instability and/or airways obstruction, patients should be urgently submitted to bronchoscopic procedures without assessing the patient under CT scan [45]. First of all, airways must be immediately protected with endotracheal intubation (ETI) by positioning either ETT or RB [4,43,44,45]. RB allows for more efficient suctioning of blood clots which leads to better airway visualization. Bronchial blockers can be used via rigid or flexible bronchoscopy to tamponade bleeding sites and preventing spillage of blood into the non-bleeding lung. Bronchial blockers may also achieve stable one-lung ventilation until definitive treatment is offered. Endoscopic therapies such as laser, electrocautery, and argon plasma coagulation can be safely performed through RB to control bleeding from visualized endobronchial lesions. The insertion of flexible bronchoscope into ETT or RB is of help for reaching more peripherally the bronchial tree and being more effective in controlling massive bleeding. FBO and RB are very effective to manage respectively peripheral (i.e., blocker balloon, tamponade, spigot) and central bleeding (i.e., argon-plasma coagulation, laser) [45]. Severe bleeding of endoscopically visible bronchogenic carcinoma could be successfully treated with interventional bronchoscopic procedures [3,4,13]. Although in the clinical practice both endobronchial administration of adrenaline and other substances and bronchial positioning of balloon catheters have been largely employed for controlling massive bleeding, no scientific data support these empirical remedies [1,14,46,47,48]. Intubating selectively the unaffected lung with single or double-lumen ETT [49,50] could be driven by FBO in order to buy time to find the way to stop the bleeding in the affected lung. FBO may be also useful in invasively ventilated patients who developed haemoptysis secondary to massive alveolar hemorrhage [4,51,52,53].

### 2.5. Atelectasis

Even if FBO is routinely use in acute patients to treat atelectasis in non-ventilated and intubated patients, the evidence in favor of this practice is surprisingly poor [54]. Literature data based on small series of invasively ventilated patients [55,56,57,58] reported a success rate of FBO in resolving atelectasis ranging between 19–89%, showing higher values in case of lobar as compared to sub-segmental atelectasis. According to few old studies, it seems that BAL or high-pressure insufflations though a balloon-tipped catheters could provide further higher likelihood of success to solve out bronchial mucous plug [56,58]. Two small RCTs [59,60] conducted in IMV patients showed similar effectiveness of bronchoscopic procedures as compared to intensive chest-physiotherapy in terms of prevention and treatment of post-lobectomy atelectasis. In consideration of the scanty proofs in favor of FBO in critically ill patients with lung atelectasis, FBO should be reserved to very selected intubated patients who develop lobar atelectasis despite intensive physiotherapy [1].

### 2.6. Central Airway Obstruction 

Interventional bronchoscopy has to be performed at the earliest timing to manage CAO due to benign or malignant diseases in spontaneous breathing patients, with the aim of avoiding IMV. Interventional procedures lead to quick symptom relief and may work as a bridge for further etiologic therapy [13]. In patients admitted to ICU/RHDCU for CAO of different etiologies necessitating IMV, uncontrolled data suggested that RB treatment, such as ablative procedures and/or airway stenting, are feasible and safe; these procedures could also facilitate weaning from ventilation, prolong survival, reduce hospital stay, as well as allow additional cancer treatments, and palliative care [61,62,63].

### 2.7. Inhaled Foreign Body 

Tracheobronchial FB aspiration is an event that occurs less commonly in adults as compared to children [64]. Nevertheless, FB may become a pulmonary emergency in adults at high risk of accidental inhalation because of dysphagia, neurological diseases, and history of excessive consumption of alcohol and psycho-neurologic drugs [65]. When there is a suspect of FB, the tracheobronchial tree should be quickly examined with FBO. The oral route is preferable since FBs of larger size could not be removed off the airways through the nasal route. FB removal has to be performed by an expert physician in an environment where RB is quickly available as rescue strategy in the following situations: acute CAO, unsuccessful FBO attempts, bleeding, FB displacement, and requirement of mechanical ventilation [65]. During FB extraction, special care should be taken in order to avoid pushing FBs more peripherally out of the endoscopic view, leaving surgery as the only effective option. A potential lethal complication is the occurrence of complete CAO due to the dislocation of the whole or part of the FB into the contra-lateral bronchial side or at tracheal site. Whenever possible, FB extraction should be performed using RB. This is because RB has the advantage of allowing the full protection of the airway and a better management of FBs thanks to the availability of special forceps or other endobronchial devices [66]. The use of FBO through RB is a successful strategy for finding more distal FBs keeping an optimal airway protection and ventilator control. In the opposite, FBO is preferable to RB in patients requiring IMV, or in those with spine/craniofacial fractures when manipulations required for RB are contraindicated [65]. The success rate of FBO extraction by means of bronchoscopy in adults varies between 60 and 90% [64,65,66]. In intubated and ventilated patients, the approach is similar for what has been reported for nonventilated patients managed by means of RB; FBO is inserted though ETT to localize and remove FB.

### 2.8. Difficult Intubation, Support to Tracheostomy, and Other Applications

Intubation with FBO (FOI) plays a key role for non-urgent management of difficult airways in patients who are awake and spontaneously breathing [5,67,68,69]. While the success rate in ETI is similar with FOI as compared to video-laryngoscopy, the time of intubation is longer and the likelihood of hypoxemia is higher with the former [70,71,72]. In some specific clinical circumstance, such as cervical-spine injury, FOI is favored over other intubation-facilitating techniques for some issues such as identification of a coexistent airway trauma, assurance the protective position of head and neck, lowering the occurrence of aspiration, and avoidance of complications due to incorrect ETT placement [73,74]. FOI has higher chances of success and safety if the clinician has enough time for a set-up, the patient is kept cooperative and in spontaneous breathing, and if the team has good FBO expertise. Drawbacks of FOI in following acute situations have to be carefully taken into consideration: it is not indicated in emergency because it is not a short time procedure, thus it is not safe in the “cannot intubate/cannot ventilate” scenario; it needs a long time experience in FBO handling; it requires techniques assuring good oxygenation and ventilation during the maneuver (i.e., manual bag ventilation, HFNC, NIV, laryngeal mask airway); it is likely to fail with deeper degree of analgo-sedation due to airway collapse [69].

FBO-guided PDT is favored over the “blinded” maneuver, because the former is associated with a lower risk of pneumothorax, cannula mal-placement, and injury of tracheal back-wall [6]. Further strengths of FBO-driven PDT are prevention of accidental extubation, toilet of secretions, and management of endobronchial hemorrhage [6,75]. Conversely, some pitfalls should be considered: (1) hypercarbia favoring increase in endo-cranial pressure in patients with central nervous system diseases, (2) risks of bleeding or incorrect cannula placement in difficult neck anatomy, coagulative disorders, overweight, and history of previous tracheostomy, (3) accidental bronchoscope damage [2].

Other applications of FBO in ICU are: working guide for inserting double-lumen endo-tracheal tubes, bronchial blockers, devices and stents to manage CAO and massive haemoptysis; assessment of pharyngeal airway swelling, vocal cord function, and subglottic stenosis is patients with risky extubation [76].

### 2.9. Complications of Bronchoscopy in Non-Ventilated Patients

FBO may have relevant negative cardio-pulmonary effects in spontaneously breathing patients [1,4,77]. FBO determines a reduction of tracheal lumen of 10–15% with a consequent increase in airway resistances and “air-trapping”, worsening of blood gases and work of breathing. FBO-induced hypoxemia is estimated in a drop of arterial oxygen pressure (PaO_2_) between 10 and 20 mmHg; this effects is aggravated after BAL due to ventilation–perfusion mismatch and during suction due to end-expiration alveolar closure [1,4,77,78]. Hypercarbia may be triggered or worsened by the use of analgo-sedation during FBO [79]. Changes in blood gases persist after the procedure and are fully reversible after a variable gap time ranging from 20 min in healthy persons to more than 24h hours in subjects with cardio-pulmonary diseases [80]. Moreover, bleeding and pneumothorax may complicate bioptic sampling and may trigger/worsen severe ARF [1,79]. FBO could increase heart rate and cardiac output as a consequence of sympathetic stimulation, hypoxemia, and decreased intra-thoracic pressure. The latter mechanism causes a rise in right ventricular pre-load and left ventricular after-load. Other cardiovascular complications are arrhythmias, and, at lower incidence rate, acute heart failure and acute myocardial ischaemia [81]. These cardio-pulmonary variations may be much more evident during RB vs. FBO procedures because of greater size of the instrument, longer time needed for suctioning, lower fraction of inspiratory O_2_ during laser treatment, and deeper analgo-sedation [82].

Data about the clinical outcomes of these FBO induced cardio-pulmonary physio-pathologic effects in hypoxemic non-ventilated patients are contrasting [77,78]. The rate of intubation in critically ill cancer patients undergoing BAL was similar as compared to that observed in those managed with strategies based on “non-invasive” diagnostic tests [83]. In a large ICU prospective study, one third of the FBO procedures were complicated by escalating ventilatory support in 169 non-intubated hypoxemic patients (i.e., NIV after “oxygen-support” failure and IMV after NIV-support failure). While the intubation rate was reported in 15% over time in the overall population, the need of IMV within 2 hours after FBO was observed in only 4% of the patients [84]. Even in absence of supporting clinical evidence, ATS guidelines did not recommend FBO with BAL in hypoxemic patients receiving supplemental oxygen for values of PaO_2_ lower than 75 mm Hg and/or an oxygen saturation (SpO_2_) lower than 90% [1,85]. Conversely, the choice of intubating an hypoxemic patient just to perform safely FBO procedures in the absence of mandatory criteria for IMV is associated with a risk of severe ETI and IMV-correlated complications (i.e., baro/volutrauma, VAP, prolonged weaning) [1,77].

### 2.10. Bronchoscopy and Noninvasive Respiratory Support 

A strong rationale supports the combined use of FBO and NIV in ARF patients as the pitfalls of each of the single procedure is offset by the physiologic effects of the other one [77,86]. FBO-induced cardiopulmonary complications may be prevented by the favorable effects of NIV in supporting and unloading respiratory muscles, correcting hypoxemia and hypercapnia, improving heart performance. Conversely, the risk of NIV failure due to the excessive burden of accumulated secretions may be prevented by the capability of FBO of clearing the airways under NIV [77]. 

Literature data on the combined use of NIV (included CPAP) and FBO mostly involve the application of the former to prevent the risk of blood gases derangement in spontaneously breathing ARF patients who have to undergo diagnostic FBO [87,88,89,90,91,92,93]. Most of the published data include uncontrolled studies that largely varies for several issues: pattern and degree of ARF, comorbidities, environment, modality of ventilator support, type of mask, route of FBO access during NIV, and bronchoscopic techniques [86]. In two uncontrolled studies [94,95], FBO with BAL was performed in patients already supported by NIV because of moderate–severe hypoxemia with a PaO_2_/FiO_2_ ratio lower than 200 mmHg. Due to the greater severity of these ventilated patients, the percentage need for intubation at 48 hours after bronchoscopy was greater than that observed in a series of less severe patients receiving NIV only during FBO (39–45% vs. 0–11%). 

One case-control study [96] performed in COPD patients with hypercapnic encephalopathy and abundant secretions showed that the strategy based on the combined use of early therapeutic FBO as toilet and NIV was as effective as the strategy based on FBO after IMV in terms of improvement of blood gases and acidosis, hospital mortality, and durations of hospitalization and ventilation. These findings support this NIV plus FBO therapeutic option versus IMV plus FBO strategy in selected COPD patients; however, further larger and controlled studies are needed to confirm these preliminary results. 

Other potential applications of the combined use of FBO and NIV in critical patients are correlated to FOI in two challenging scenarios, difficult airways and NIV failure management [97,98,99]. 

Negative pressure ventilation delivered by a poncho-wrap to support RB procedures under general anesthesia was associated with better important clinical outcomes such as hypoventilation with need for assisted manual ventilation, higher inspiratory oxygen fraction, opioids doses, and length of recovery, as compared to alternatives strategies (i.e., assisted spontaneous breathing and external high-frequency oscillation) [100,101]. It should be considered that these results obtained by only one expert center have not been replicated elsewhere.

Expertise of the team of ICU/RHDCU in dealing with NIV, FBO, and cardiopulmonary emergencies are the keys point for achieving the success of the combined use of NIV and FBO [86]. The following points have been suggested: spontaneous breathing patients have to be adapted to NIV by 20 min before FBO and kept within 30–90 min after the procedure; NIV should be initially set with low levels of pressure support (i.e., 10 cmH_2_O) and PEEP (i.e., 5 cmH_2_O) with titration of pressure support in order to obtain an expiratory tidal volume of 8–10ml/kg and respiratory rate lower than 25 min; air leaks have to be minimized to prevent hypoventilation; oro-facial or total face masks are preferable for NIV–FBO procedures; the choice of oral vs nasal access of bronchoscope to the airways depends on the handling expertise of the team [77]; it is recommended to provide adequate topical anesthesia and mild analgo-sedation to facilitate the NIV plus FBO procedure. Complications of the combined NIV plus FBO strategy should be carefully considered and are dependent both on NIV and FBO itself: abdomen distension, aspirative syndrome, over and under patients respiratory support, and skin damages are correlated to the former while cardiovascular risks, blood gases derangement, pneumothorax, and bleeding are correlated to the latter; finally, analogo-sedation should be carefully titrated to prevent drug related complications [86].

Similar to what was reported for NIV, favorable synergistic effects may be expected from the combined use of FBO and HFNC in acute critically ill patients. HFNC has been shown to give physiologic and clinical advantages over conventional oxygen therapy in terms of delivery of high and reliable inspiratory oxygen faction supply, good humidification, expectoration facilitation, great subject comfort, provision of 3–6 cmH_2_O of PEEP values, alveolar recruitment, wash out of CO2, and unload of respiratory muscles [102]. According to few RCT performed in critically hypoxemic patients undergoing diagnostic FBO, HFNC turned out to be better than oxygen therapy, but less efficient than NIV, in terms of oxygenation indexes during and after the procedure [103,104]. From a practical point of view, FBO procedures are easier to perform and better tolerated under HFNC as compared under NIV in ARF patients.

### 2.11. Bronchoscopy during IMV

Performing FBO in intubated and invasively ventilated patients produces cardiopulmonary changes which are similar to what was observed in spontaneously breathing patients [2]. The amount of FBO-induced cardiopulmonary repercussions is strictly dependent on the difference existing between the external diameter of the bronchoscope and the internal diameter of the endotracheal tube or tracheal cannula. In non intubated subjects, during FBO, the cross-sectional area of the trachea is reduced by 10–15% due to the presence of the instrument. Conversely, during broncoschopic procedures performed in IMV patients, this tracheal area is reduced by 40% when a bronchoscope with the external diameter of 5.7 mm is inserted through an ETT with an internal diameter of 9 mm ETT, and by 66% if the subject is intubated with a 7-mm ETT. If the physicians do not care about this key point, there is an increased risk of providing an inadequate ventilation with “air-trapping” and of damaging the bronchoscope [1]. Recommendations in performing FBO procedures in invasively ventilated patients are: pre-oxygenation with O_2_ delivered at 100% as inspiratory fraction during and immediately after the maneuver; setting the ventilator in mandatory modalities, ensuring adequate tidal volume and respiratory rate and avoiding triggered modes, and access of the bronchoscope though a special swivel connector with a perforated diaphragm that avoids dangerous drop in PEEP/CPAP, with the risk of alveolar de-recruitment in severe ARDS patients [1]. 

## 3. Conclusions 

Bronchoscopy covers an important diagnostic and therapeutic role in ARF patients. The balance between the benefits and the potentially life-threatening complications of bronchoscopy should be considered, provided that drawbacks and indications of bronchoscopic procedures in ventilated, both invasively and non-invasively, and in spontaneously breathing patients are clearly understood. Performing bronchoscopic procedures in respiratory critically ill subjects requires an adequate monitoring of patients, the capability to manage urgent airways difficulties, and cardio-pulmonary complications. In ICU/RUDCU settings, bronchoscopy may be of help in the management of difficult airways (i.e., intubation and tracheostomy), in the diagnostic flow chart of lung infiltrates in spontaneously breathing and ventilated patients with suspected pneumonia or ILD, in the treatment of severe CAO and massive bleeding. The combined use of either NIV or HFNC during bronchoscopy has been reported as a successful strategy in different diagnostic and therapeutic clinical conditions; currently, NIV-supported during FBO in non-ventilated hypoxemic patients to prevent blood gas deterioration and eventual ETI is the scenario with the highest level of evidence.
